# Spoligotyping of *Mycobacterium africanum*, Burkina Faso

**DOI:** 10.3201/eid1801.110275

**Published:** 2012-01

**Authors:** Michel K. Gomgnimbou, Guislaine Refrégier, Serge P. Diagbouga, Sanou Adama, Antoinette Kaboré, Adama Ouiminga, Christophe Sola

**Affiliations:** CNRS–Université Paris–Sud, Orsay, France (M.K. Gomgnimbou, G. Refrégier, C. Sola);; Ministère de la Santé, Ouagadougou, Burkina Faso (S.P. Diagbouga);; Centre Muraz, Bobo Dioulasso, Burkina Faso (S. Adama, A. Kaboré, A. Ouiminga)

**Keywords:** Mycobacterium africanum, Burkina Faso, spoligotyping, bacteria, tuberculosis and other mycobacteria

## Abstract

Using Ziehl-Neelsen–positive slides collected from tuberculosis diagnostic centers in Burkina Faso, we showed that 20% of 80 spoligotyping-positive DNA samples had a characteristic *Mycobacterium africanum*–specific genomic signature. This result suggests that *M. africanum* is still present in Burkina Faso at almost the same prevalence as 15–20 years ago.

*Mycobacterium africanum* remains a major pathogen in Africa (*1*). Recently, de Jong et al. estimated the prevalence of *M. africanum* to be 1.7% in Burkina Faso (*1*); their estimate was based on a 2007 study by Godreuil et al., who unexpectedly did not identify any *M. africanum* isolate within a collection of 120 *M. tuberculosis* complex clinical isolates in 2001 from 79 tuberculosis patients living in Ouagadougou and 41 living in Bobo Dioulasso, the 2 largest cities in the country (*2*). However, 2 patterns (isolates 94 and 90) can be recognized as *M. africanum* by their characteristic spoligotyping signature (deletion of spacers, 8, 9, and 39) and an association of mycobacterial interspersed repetitive unit (MIRU) 24 >2, MIRU31 >4, and MIRU40 <3 signature (*2**,**3*).

In the neighboring country of Ghana (which has 200 km of common borders with Burkina Faso), another study suggested that the population structure of *M. tuberculosis* complex comprises 1) 34% spoligo-international type (SIT) 61 (named the Cameroon clade, also present in Burkina Faso); 2) 30% *M. africanum* (including *M. africanum* West African 1 and West African 2); and 3) 36% principal genetic group 2 and 3 modern strains (e.g., T, U [unknown], Haarlem, X, LAM [Latino-American and Mediterranean]), with minor prevalence of other principal genetic groups, i.e., the East-African Indian, Beijing, and *M. bovis* clades (*4**,**5*). These observations—and their congruence to estimates by Ledru et al. in 1996 of an 18.4% prevalence of *M. africanum* strains isolated from the 300 patients in whom tuberculosis was newly diagnosed in Burkina Faso during 1992–1994 (*5*)—prompted us to reexamine the conclusions of Godreuil et al. on the *M. africanum* prevalence in Burkina Faso.

## The Study

The study, which we conducted during March–September 2010, had 3 goals. First, we wanted to determine whether we could extract DNA and perform high-throughput spoligotyping on a Luminex 200 device (Luminex, Austin, TX, USA) on acid-fast bacillus–positive slides (*6*). Second, we wanted to reestimate the prevalence of *M. africanum* in Burkina Faso from a recent and random sample of slides. Third, we wanted to further analyze the relative proportion of *M. africanum* West African I and West African 2 strains in Burkina Faso because this country is part of central western Africa, where the 2 *M. africanum* West African 1 and 2 strains are present at various relative rates (*2*). We report on all the goals of this project, even though goal 3 remains to be confirmed because of the small sample size.

From within 14 geographically independent centers in Burkina Faso (Figure), we recruited a random sample of 186 Ziehl-Neelsen (ZN) slides that had been included in a national study on drug resistance, as approved by the ethical committee for health research in Burkina Faso (2007–031; June 28, 2009). Of 186 DNA samples extracted from as many ZN slides, 143 sputum samples had been scored 3+, 18 were scored 2+, 10 were scored 1+, 5 had 1–9 bacilli total (±), totaling 176 positive slides from as many sputum samples. In addition, test results were negative for 9 and unknown for 1.

**Figure:**
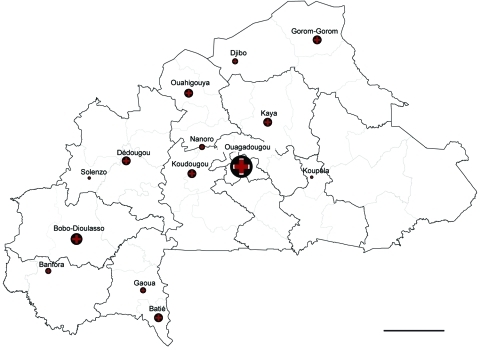
Origin of samples described in study of *Mycobacterium africanum* in Burkina Faso. Dark outlined borders indicate province; light outlined borders indicate regions. Scale bar = 100 km. Sources: Institut Géographique du Burkina Faso/Centre Muraz-PNT.

In a preliminary trial of 9 independent 3+ positive slides, DNA extraction was attempted by 2 methods: an enzymatic method (*7*) and a classical thermic lysis in a Chelex suspension (InstaGene; Bio-Rad, Hercules, CA, USA) (*8*). In our study, only the Chelex method produced good results, i.e., enabled us to obtain DNA that was successfully PCR amplified and produced a full spoligotyping pattern (results not shown). The quantity of DNA extracted was superior for all tests by the enzymatic lysis (n = 3) as by the Chelex (n = 6), as estimated by spectrophotometry (NanoDrop ND-1000; LabTech, Ringmer, UK). Thus, DNA can be successfully extracted by the enzymatic method for many human or bacterial cells but not for *M. tuberculosis* complex because no spoligotype could be obtained. We therefore analyzed the 176 experimental slides by using the Chelex extraction procedure.

The origins of all ZN slides assessed in this study are shown in the Figure, and genotyping results are shown in the Table (full results in the Technical Appendix). As observed in a much larger set of samples from Ghana, the Cameroon family (SIT61) also prevails in Burkina Faso (18 [25%] isolates) (*4*). Whether the Cameroon strains from Burkina Faso are similar or identical to those from Ghana remains to be studied.

**Table:** Distribution of classified genotypes of *Mycobacterium tuberculosis* complex, Burkina Faso*

Clade†	No. (%) isolates, n = 72‡
CAM	18 (25)
Including CAM_family prototype = SIT61	14 (19)
Other CAM	4 (6)
T	16 (22.2)
Including T1	10 (13.9)
Undefined T1-T2	2 (2.8)
T2	1 (1.4)
T3	1 (1.4)
T5_MAD2	2 (2.8)
Haarlem	10 (13.9)
Including H1	7 (9.7)
H3	3 (4.2)
X	4 (5.6)
Including X3	1 (1.4)
*M. africanum* I (WA I and WA 2)	16 (22.2)
*M. bovis*	1 (1.4)
CAS1_Delhi	5 (6.9)
LAM9	1 (1.4)
Beijing	1 (1.4)

*CAM, Cameroon; SIT, spoligo-international type; WA, West African; CAS, Central Asian; LAM, Latino American–Mediterranean. †Described in (*9*). ‡Excludes 4 new and 4 unclassified genotypes.

Our result confirms the observation by Godreuil et al. in 2007 on the prevalence of the SIT61/Cameroon strains in Burkina Faso (*2*). However, we detected 16 *M. africanum* spoligotypes (West African 1 and West African 2), i.e., a minimal *M. africanum* prevalence of 20%, close to 18.4% found by Ledru et al. in 1996 (*5*). Third, the T and Haarlem strains represented 16 (22%) and 10 (14%), respectively, of the patterns; other genotypes were rare (5 CAS [Central Asian], 4 X, 1 *M. bovis*, 1 Beijing, 1 LAM). Finally, the relative prevalence of *M. africanum* West African 1 from *M. africanum* West African 2 could first be assessed by the spoligotyping signature (2 vs. 14; Technical Appendix). Specific single nucleotide polymorphism detection could constitute another classification tool for *M. africanum* sublineages (*10**,**11*). Unfortunately, detection of katG203 single nucleotide polymorphism failed on the slide-extracted DNAs (results not shown), and our study is limited by a suboptimal yield in positive spoligotyping results (80 [43%] of 186), an issue that should be improved.

## Conclusions

The results of our study diverge on the *M. africanum* prevalence in Burkina Faso from results from Godreuil et al. (*2*) (1.9% vs. 20%). These authors were intrigued to not identify more *M. africanum* isolates and suggested that their finding might reflect “a decrease in *M. africanum* prevalence in these countries,” referring to a similar decrease in Cameroon during 1971–2003 (*12**,**13*). We believe that in the study by Godreuil et al., an unintentional bias was introduced against *M. africanum*, given the difficulty of isolating this genotypic variant in routine practice in mycobacteriologic laboratories. Differences in *M. africanum* prevalence in culture-based and sputum-based studies might reflect the difficulties of growing and isolating *M. africanum* in some national TB reference laboratories in western Africa. *M. africanum*, which is closely related to *M. bovis*, has peculiar growing requirements that are not always satisfied. Supplementation of Löwenstein-Jensen medium with pyruvate is mandatory and not standardized (from 0.1% to 0.4%).

The pyruvate requirements of some members of the *M. tuberculosis* complex were recently shown to be caused by a mutation creating an inactive pyruvate kinase (*14*). This specific mutation of *M. africanum* has major implications for its metabolism and growth.

Implementation of adequate culture and molecular identification facilities in Burkina Faso are needed. A potential solution to avoiding the bias from culture and from DNA extraction from slides could be to extract DNA directly from sputum, e.g., by storing surplus sputum prospectively in 70% ethanol. Additional work also is needed to improve analytical methods for ZN slides to refine description of *M. tuberculosis* genetic diversity and eventually to provide predictive genetic drug susceptibility testing. Introduction of newer and faster TB diagnostic methods are urgently needed in this area of western Africa**.**

## Supplementary Material

Technical Appendix Spoligotyping of *Mycobacterium africanum*, Burkina Faso.
