# Supplementary material for: Spoligotyping of Mycobacterium africanum, Burkina Faso
Source: Emerg Infect Dis. 2012 Jan;18(1):117–9. doi: 10.3201/eid1801.110275 (PMC3310091; doi:10.3201/eid1801.110275)
Supplement: Technical Appendix — Spoligotyping of Mycobacterium africanum, Burkina Faso. [file 11-0275-Techapp_2p.pdf]

Article DOI: http://dx.doi.org/10.3201/eid1801.110275

Spoligotyping of *Mycobacterium africanum*, Burkina Faso

Technical Appendix

SIT*	No.	Spoligo binary	Clade	Geographic origin	Center
ND	137C (3+)	■■■■■□■□□□□□■■■■■■■■□□□□■■■■■■■■■■■■□□□■■■■	West African 1	unknown	
ND	108B (3+)	■■■■■■□□□□□□□□□□■■■■□□□□■■■■■■■■■■■■□□□■■■■	West African 1	Ouagadougou	CNLAT
ND	028B (3+)	■■■■■■□□□■■■■■□■■■■■■□□□■■■■■■■■■■■■■■□■■■■	West African 2	Gorom Gorom	Gorom Gorom
181	049A (3+)	■■■■■■□□□■■■■■■■■■■■■■■■■■■■■■■■■■■■■□■■■■	West African 2	Gorom Gorom	Gorom Gorom
181	059A (3+)	■■■■■■□□□■■■■■■■■■■■■■■■■■■■■■■■■■■■■□■■■■	West African 2	Dédougou	Dédougou
187	080A (3+)	■■■■■■□□□■■■■■■■■■■■■■■■■■■■■■■□■■■■■□■■■■	West African 2	Bobo Dioulasso	CRLAT
ND	095B (3+)	■■■■■■□□□■■■■□■■■■■■■□□□■■■■■■■■■■■■■■□■■■■	West African 2	Kaya	Kaya
326	133A (3+)	■■■■■■□□□■■■■■■■■■■■■□□□■■■■■■■■■■■■■■□■■■■	West African 2	Ouagadougou	CNLAT
326	177A (3+)	■■■■■■□□□■■■■■■■■■■■■□□□■■■■■■■■■■■■■■□■■■■	West African 2	Gaoua	Gaoua
ND	234B (3+)	□■■■■■□□□■■■■■□■■■■■■■■■■■■■■■■■■■■■■□■■■■	West African 2	Bobo Dioulasso	CRLAT
326	280A (3+)	■■■■■■□□□■■■■■■■■■■■■□□□■■■■■■■■■■■■■■□■■■■	West African 2	Banfora	Banfora
326	318A (3+)	■■■■■■□□□■■■■■■■■■■■■□□□■■■■■■■■■■■■■■□■■■■	West African 2	Dédougou	Dédougou
181	403A (3+)	■■■■■■□□□■■■■■■■■■■■■■■■■■■■■■■■■■■■■□■■■■	West African 2	Nanoro	Nanoro
ND	531A (3+)	□□□□□□□□□□□□□□□□□□■■■□□□■■■■■■■■■■■■■■□■■■■	West African 2	Ouagadougou	Paul VI
187	548A (3+)	■■■■■■□□□■■■■■■■■■■■■■■■■■■■■■■□■■■■■□■■■■	West African 2	Bobo Dioulasso	CRLAT
326	594C (3+)	■■■■■■□□□■■■■■■■■■■■■□□□■■■■■■■■■■■■■■□■■■■	West African 2	Dédougou	Dédougou
1	027A (3+)	□□□□□□□□□□□□□□□□□□□□□□□□□□□□□□□□□□■■■■■■■■■	Beijing	Batié	Batié
991	271A (3+)	■■□□□■■■□■■■■■■□■■■■■■□■■■■■■■■■■■■■■■□□□□□	*M. bovis* 1	Ouagadougou	CMA Pissy
ND	010B (3+)	■■■■■■■■■■■■■■□■■■□□■■□□□■■■■■■■□□□□■■■■■■■	CAM	Bobo -Dioulasso	CMA Dafra
61	020A (3+)	■■■■■■■■■■■■■■■■■■■■■■□□□■■■■■■■□□□□■■■■■■■	CAM	Banfora	Banfora
61	026B (3+)	■■■■■■■■■■■■■■■■■■■■■■□□□■■■■■■■□□□□■■■■■■■	CAM	Batié	Batié
61	084A (3+)	■■■■■■■■■■■■■■■■■■■■■■□□□■■■■■■■□□□□■■■■■■■	CAM	Ouagadougou	CMA Pissy
ND	087A (3+)	□■■■■■■■■■■■■■■■■■■■■■□□□■■■■■■■□□□□■■■■■■■	CAM	Ouahigouya	Ouahigouya
61	094A (3+)	■■■■■■■■■■■■■■■■■■■■■■□□□■■■■■■■□□□□■■■■■■■	CAM	Nanoro	Nanoro
61	112A (3+)	■■■■■■■■■■■■■■■■■■■■■■□□□■■■■■■■□□□□■■■■■■■	CAM	Batié	Batié
61	180B (3+)	■■■■■■■■■■■■■■■■■■■■■■□□□■■■■■■■□□□□■■■■■■■	CAM	Ouagadougou	CNLAT
61	204B (3+)	■■■■■■■■■■■■■■■■■■■■■■□□□■■■■■■■□□□□■■■■■■■	CAM	Kaya	Kaya
61	278A (3+)	■■■■■■■■■■■■■■■■■■■■■■□□□■■■■■■■□□□□■■■■■■■	CAM	Koudougou	Koudougou
61	426A (3+)	■■■■■■■■■■■■■■■■■■■■■■□□□■■■■■■■□□□□■■■■■■■	CAM	Banfora	Banfora
61	474A (3+)	■■■■■■■■■■■■■■■■■■■■■■□□□■■■■■■■□□□□■■■■■■■	CAM	Gaoua	Gaoua
61	420A (3+)	■■■■■■■■■■■■■■■■■■■■■■□□□■■■■■■■□□□□■■■■■■■	CAM	Ouagadougou	CNLAT
61	580A (3+)	■■■■■■■■■■■■■■■■■■■■■■□□□■■■■■■■□□□□■■■■■■■	CAM	Ouagadougou	CMA Pissy
57	592C (3+)	■■■■■■■■■■■■■■■■■■□□■■□□□■■■■■■■□□□□■■■■■■■	CAM	Ouagadougou	CNLAT
2550	602A (3+)	■■■■■■■■■■■■□■■■■■■■■■□□□■■■■■■■□□□□■■■■■■■	CAM	Bobo Dioulasso	CRLAT
61	605A (3+)	■■■■■■■■■■■■■■■■■■■■■■□□□■■■■■■■□□□□■■■■■■■	CAM	Bobo Dioulasso	CRLAT
61	611A (3+)	■■■■■■■■■■■■■■■■■■■■■■□□□■■■■■■■□□□□■■■■■■■	CAM	Ouahigouya	Ouahigouya
1327	079B (3+)	■■■□□□□■■■■□□■■■■■■■■■□□□□□□□□□□□□□■■■■■■■■	CAS1_Delhi	Bobo Dioulasso	CRLAT
ND	085A (3+)	■■■□□□□■■■■□□■□■■■■■■■□□□□□□□□□□□□□■■■■■■■■	CAS1_Delhi	Djibo	Djibo
ND	129A (3+)	■■■□□□□■■■■■■■□■□□□□□□□□□□□□□□□□□□□■■■■■■■	CAS1_Delhi	Djibo	Djibo
1327	188A (3+)	■■■□□□□■■■■□□■■■■■■■■■□□□□□□□□□□□□□■■■■■■■■	CAS1_Delhi	Ouagadougou	CMA 30
1327	481A (3+)	■■■□□□□■■■■□□■■■■■■■■■□□□□□□□□□□□□□■■■■■■■■	CAS1_Delhi	Ouagadougou	CNLAT
47	038B (3+)	■■■■■■■■■■■■■■■■■■■■■■■■■□□□□□□■□□□□■■■■■■■	H1	Djibo	Djibo

SIT*	No.	Spoligo binary	Clade	Geographic origin	Center
47	054A (3+)	■■■■■■■■■■■■■■■■■■■■■■■■■□□□□□□■□□□□■■■■■■■	H1	Nanoro	Nanoro
47	099B (3+)	■■■■■■■■■■■■■■■■■■■■■■■■■□□□□□□■□□□□■■■■■■■	H1	Ouagadougou	CMA30
47	132B (3+)	■■■■■■■■■■■■■■■■■■■■■■■■■□□□□□□■□□□□■■■■■■■	H1	Ouagadougou	CNLAT
47	386A (3+)	■■■■■■■■■■■■■■■■■■■■■■■■■□□□□□□■□□□□■■■■■■■	H1	Ouagadougou	CNLAT
47	397A (3+)	■■■■■■■■■■■■■■■■■■■■■■■■■□□□□□□■□□□□■■■■■■■	H1	Koupéla	Koupéla
47	583B (3+)	■■■■■■■■■■■■■■■■■■■■■■■■■□□□□□□■□□□□■■■■■■■	H1	Koudougou	Koudougou
50	535A (2+)	■■■■■■■■■■■■■■■■■■■■■■■■■■■■■■□■□□□□■■■■■■■	H3	Solenzo	Solenzo
49	606C (3+)	■■■■■■■■■■■■■■■■■■■■■■■■■■■■■■□■□□□□■■■□■■■	H3	Dédougou	Dédougou
50	609A (3+)	■■■■■■■■■■■■■■■■■■■■■■■■■■■■■■□■□□□□■■■■■■■	H3	Ouahigouya	Ouahigouya
42	522A (3+)	■■■■■■■■■■■■■■■■■■■■□□□□■■■■■■■■□□□□■■■■■■■	LAM9	Gaoua	Gaoua
ND	206A (3+)	■■■■■■■■■■■■■■□■■■■■■■■■■■□□□□□□□□□□□■■■■■■	New	Batié	Batié
ND	232B (3+)	■■■■■□□□■■■■■■■■■□□□■■■■■■■■■■■■□□□□■■■■■■■	New	Solenzo	Solenzo
ND	455A (3+)	■■■■■■■■■■■■□■□■■■■□■■□■■■■■■■■■□□□□■■■■■■■	New	Koudougou	Koudougou
ND	589B (3+)	■■■■■■■■■■■■■■■■■■■■■□□□□□□■■■■■□□□□■■■■■■■	New	Gorom Gorom	Gorom Gorom
53	051A (3+)	■■■■■■■■■■■■■■■■■■■■■■■■■■■■■■■■□□□□■■■■■■■	T1	Ouagadougou	CMA 30
462	187A (3+)	■■■■■■■■■■■■■■■■■■■■■■■■■■■■□■■■□□□□■■■■■■■	T1	Ouagadougou	CMA 30
53	438A (IC)	■■■■■■■■■■■■■■■■■■■■■■■■■■■■■■■■□□□□■■■■■■■	T1	Kaya	Kaya
53	480C (3+)	■■■■■■■■■■■■■■■■■■■■■■■■■■■■■■■■□□□□■■■■■■■	T1	Ouagadougou	CNLAT
53	495A (2+)	■■■■■■■■■■■■■■■■■■■■■■■■■■■■■■■■□□□□■■■■■■■	T1	Ouagadougou	PPH Yalgado
804	533B (3+)	■□□■■■■■■■■■■■■■■■■■■■■■■■■■■■■■□□□□■■■■■■■	T1	Koudougou	Koudougou
53	534B (3+)	■■■■■■■■■■■■■■■■■■■■■■■■■■■■■■■■□□□□■■■■■■■	T1	Ouagadougou	PPH Yalgado
53	547A (3+)	■■■■■■■■■■■■■■■■■■■■■■■■■■■■■■■■□□□□■■■■■■■	T1	Bobo Dioulasso	CRLAT
53	549A (3+)	■■■■■■■■■■■■■■■■■■■■■■■■■■■■■■■■□□□□■■■■■■■	T1	Bobo Dioulasso	CRLAT
53	607A (3+)	■■■■■■■■■■■■■■■■■■■■■■■■■■■■■■■■□□□□■■■■■■■	T1	Kaya	Kaya
78	062A (3+)	■■■■■■■■■■■■■■■■■■■■■■■■■■■■■■■■□□□□■■■□□■■	T1-T2	Bobo Dioulasso	CMA Dafra
78	601A (3+)	■■■■■■■■■■■■■■■■■■■■■■■■■■■■■■■■□□□□■■■□□■■	T1-T2	Bobo Dioulasso	CRLAT
52	585B (3+)	■■■■■■■■■■■■■■■■■■■■■■■■■■■■■■■■□□□□■■■□■■■	T2	Ouagadougou	CMA 30
504	582B (3+)	■■■■■■■■■■■■□■■■■■■■■■■■□■■■■■■■■■■□□□□■■■■■■■	T3	Koudougou	Koudougou
1227	078A (3+)	■■■■■■■■■■■■□■■■■■■□■■□■■■■■■■■■□□□□■■■■■■■	T5_MAD2	Bobo Dioulasso	CMA Dafra
1227	490A (3+)	■■■■■■■■■■■■□■■■■■■□■■□■■■■■■■■■□□□□■■■■■■■	T5_MAD2	Ouagadougou	CMA Pissy
ND	145A (3+)	■■■□□□□■■■■■■■□■■■■■■■□□■■■■■■■■■■■■□■■■■■■	U	Gorom Gorom	Gorom Gorom
1200	190B (3+)	■■■□□□□■■■■■■■■■■■■■■■□□■■■■■■■■■■■■■■■■■■■	U	Gorom Gorom	Gorom Gorom
2398	213A (3+)	■■■□□□□■■■■■■■■■■■■■■■□□■□□□□□□■■■■■■■■■■■■	U	Bobo Dioulasso	CRLAT
1200	508A (3+)	■■■□□□□■■■■■■■■■■■■■■■□□■■■■■■■■■■■■■■■■■■■	U	Ouagadougou	CNLAT
2397	159B (3+)	■■■□□□□□□□□□■■■■■□□■■■■■■■■■■■■■□□□□■■■■■■■	X3	Gorom Gorom	Gorom Gorom
2397	430A (3+)	■■■□□□□□□□□□■■■■■□□■■■■■■■■■■■■■□□□□■■■■■■■	X3	Ouagadougou	CNLAT
2397	510B (3+)	■■■□□□□□□□□□■■■■■□□■■■■■■■■■■■■■□□□□■■■■■■■	X3	Ouagadougou	CNLAT
200	608A (3+)	■■■□□□□□□□□□■■■■■□■■■■■■■■■■■■■■□□□□■■■□□□□	X3	Ouahigouya	Ouahigouya

*SIT, spoligotyping international type (*14*); CRLAT, Centre Régional de Lutte Antituberculeuse; CNLAT, Centre National de Lutte Antituberculeuse; PPH, Service de Pneumo-Phtisiologie; CMA, Centre Médical Antenne Chirurgicale.

■□

Absence
